# Predictive Performance of 2 Immunoassays in Patients with Graves Disease Undergoing Radioiodine Therapy: Prospective Study

**DOI:** 10.1210/jendso/bvaf016

**Published:** 2025-01-31

**Authors:** Marcus Vinícius Rodrigues de Souza, Marcelo Tatit Sapienza, Luciana Pinto Brito, Pedro Afonso Cortez, Suemi Marui

**Affiliations:** Disciplina de Endocrinologia, Departamento de Clínica Médica, Faculdade de Medicina, Universidade Federal de Uberlândia, Uberlândia, MG 38408-100, Brazil; Unidade de Tireoide, Disciplina de Endocrinologia e Metabologia, Hospital das Clínicas, Faculdade de Medicina, Universidade de São Paulo (HCFMUSP), São Paulo, SP 01246-903, Brazil; Divisão de Medicina Nuclear, Hospital das Clínicas, Faculdade de Medicina, Universidade de São Paulo (HCFMUSP), São Paulo, SP 05403-911, Brazil; Laboratório de Hormônios e Genética Molecular (LIM 42), Hospital das Clínicas, Faculdade de Medicina, Universidade de São Paulo (HCFMUSP), São Paulo, SP 05403-010, Brazil; Professor of Higher Education, Instituto de Psicologia, Universidade Federal de Uberlândia, Uberlândia, MG 38408-100, Brazil; Unidade de Tireoide, Disciplina de Endocrinologia e Metabologia, Hospital das Clínicas, Faculdade de Medicina, Universidade de São Paulo (HCFMUSP), São Paulo, SP 01246-903, Brazil; Laboratório de Endocrinologia Celular e Molecular (LIM 25), Hospital das Clínicas, Faculdade de Medicina, Universidade de São Paulo (HCFMUSP), São Paulo, SP 01246-903, Brazil

**Keywords:** Graves disease, thyroid-stimulating immunoglobulins, radioactive iodine, treatment, prognosis

## Abstract

**Context:**

Radioactive iodine (RAI) treatment is a well-established successful treatment for Graves disease (GD) but causes an increment in autoantibodies, particularly TSH receptor antibodies.

**Objective:**

To assess the performance and prognostic role of Immulite thyroid-stimulating immunoglobulin (TSI) and Elecsys thyrotropin receptor antibodies (TRAbs) immunoassays in patients with GD undergoing RAI therapy.

**Methods:**

Clinical and laboratory data of 188 patients (156 GD and 32 toxic nodule goiter), undergoing RAI therapy between January 2018 and January 2022 were prospectively collected over 12 months. Refractoriness was defined as persistent or recurrent hyperthyroidism 6 months post-RAI treatment without levothyroxine. Statistical analysis included descriptive statistics, logistic regression, and generalized estimated equations.

**Results:**

Patients had a mean age of 46.4 years, and 78.2% were women. RAI therapy was indicated in 94.2% due to uncontrolled hyperthyroidism or ATD therapy relapse (median of 35 months). Immulite TSI showed higher clinical sensitivity and accuracy (area under the curve [AUC]: 0.98, sensitivity 92.0%, accuracy 98.4%) than Elecsys TRAb (AUC: 0.97, sensitivity 82.1%, accuracy 91.2%). Successful treatment was achieved in 112 of 126 GD patients (89%). Thyroid volume, 2-hour iodine-131 uptake, free thyroxine and thyroxine levels, Elecsys TRAb, and Immulite TSI were significantly higher in the refractory group (*P* < 0.05), despite most patients receiving >300 Gy of RAI. Longitudinal thyrotropin evaluation predicted treatment response at 12 months (*P* = .01), whereas autoantibodies did not.

**Conclusion:**

Refractoriness to RAI therapy was associated with higher levels of Immulite TSI and Elecsys TRAb prior to treatment. Although AUCs for both assays were equivalent, Immulite TSI demonstrated superior clinical sensitivity and accuracy. Despite distinct autoantibody patterns emerging post-RAI, longitudinal monitoring did not predict treatment response after 1 year but indicated persistently high concentrations.

Graves disease (GD) is the main cause of hyperthyroidism in iodine-sufficient areas, such as Brazil, predominantly affecting females, with a peak incidence around the third to fifth decades of life [1]. The pathogenesis of this autoimmune disease involves interaction between genetic and environmental factors characterized by the production of antibodies directed against the thyrotropin receptor (TRAbs). The differentiation between stimulating (thyroid-stimulating immunoglobulin [TSI]) and blocking (thyrotrophin-binding inhibiting immunoglobulin [TBII]) forms of TRAb is carried out by bioassays, while most immunoassays measure the total pool of antibodies in the patient's serum [2]. The Immulite TSI was developed with an automated bridge technology to predominantly detect TSI and has demonstrated a high clinical value with excellent sensitivity and specificity in the diagnosis of GD [3-5].

Hyperthyroidism is treated with antithyroid drugs (ATDs), radioactive iodine (RAI) therapy, or total thyroidectomy (TT) [6]. RAI treatment is a well-established successful treatment with moderate costs compared with surgery [6, 7]. Due to radiation-induced thyrocyte destruction, an increment in autoantibodies in the first few months after RAI therapy is associated with new onset or worsening of preexisting Graves orbitopathy (GO), particularly in smokers [8]. In addition, patients treated with RAI therapy tend to sustain circulating TRAbs for longer periods than ATD treatment and TT [9, 10]. Chiovato et al suggested that an increment in TSI levels was related to the successful endpoint of RAI treatment, in contrast to persistent hyperthyroidism [11]. Fang et al found similar proportions of TSI concentrations after RAI therapy independently of the endpoint (hyperthyroidism, euthyroidism, or hypothyroidism; 40.7%, 37.5%, and 20.5%, respectively), although thyroid-blocking antibodies increased significantly in the last 2 groups (37.0%, 66.7%, and 73.5%, respectively, *P* < .01) during follow-up [12].

The objective of this study was to evaluate the performance and prognostic role of 2 immunoassays, Immulite TSI and Elecsys TRAb, in patients with GD undergoing radioiodine treatment.

## Materials and Methods

### Subjects

Patients with hyperthyroidism referred to the Nuclear Medicine Service (Clinicas Hospital, School of Medicine, University of Sao Paulo, Brazil) between January 2018 and January 2022 were recruited and prospectively studied.

The diagnosis of GD was defined by the presence of hyperthyroidism, suppressed TSH with increased free thyroxine (fT4) and/or free triiodothyronine (fT3), with diffusely increased uptake of ^131^I, high TRAb and/or TSI titers, and/or with the presence of GO. The diagnosis of toxic nodular goiter (TNG) was characterized by the presence of hyperthyroidism, undetectable TRAbs, and increased uptake of ^131^I in 1 or more nodules and detected by thyroid ultrasound [6].

Patients were excluded from the study if aged <18 years, pregnant and breastfeeding mothers, with thyroid nodules of indeterminate or malignant cytology, moderate–severe GO or inflammatory activity (clinical activity score ≥ 3), use of amiodarone, lithium, and drugs that affect thyroid function, and patients who had previously undergone thyroidectomy or RAI treatment.

This research was approved by the Ethics Committee of our Institution (CAAE: 76207417.1.0000.0068) and was conducted in accordance with the Declaration of Helsinki. All the patients gave their informed consent to take part in the study.

### Thyroid Function Tests and Antibody Assays

Blood samples were taken 7 days before RAI therapy and 3, 6, and 12 months after administration of the therapeutic dose. The samples were stored at −20 °C for simultaneous analysis using Immulite TSI and Elecsys TRAb.

TSH (RRID:AB_3095311), fT4 (RRID:AB_3095310), and fT3 (RRID:AB_2925177) were measured by the electrochemiluminescent method using the COBAS e601 analyzer, with the following reference ranges: 0.27 to 4.20 mIU/L for TSH; 11.9 to 21.8 pmol/L for fT4; and 2.76 to 6.45 pmol/L for fT3.

Thyroperoxidase antibody (TPO-Ab) was measured using an immunochemiluminometric method (RRID:AB_3674087) manufactured by Beckman Coulter (Fullerton, USA) on the Unicel DxI800 analyzer, with detection range 0.25 to 1000 IU/mL; the cutoff recommended by the manufacturer in nonpregnant adults was 9 IU/mL. Thyroglobulin antibody was measured by an immunochemiluminometric method (RRID:AB_3674086) manufactured by Beckman Coulter (Fullerton, USA) on the Unicel DxI800 analyzer, with detection range 0.9 to 2500 IU/mL; the cutoff recommended by the manufacturer in nonpregnant adults was 4 IU/mL.

Elecsys TRAb (RRID:AB_2801453) was measured by competitive chemiluminescent immunoassay (Roche Diagnostics, Mannheim, Germany) on COBAS e601 using 50 µL of the patient's serum. The method conformed to the 1st International Standardization (National Institute for Biological Standards and Control [NIBSC] 90/672), with a detection range of 0.3 to 40 IU/L; the optimal cutoff of 1.75 IU/L suggested GD diagnosis [13]. Elecsys TRAb is a competitive immunoassay that uses an immobilized purified porcine TSH-R (pTSH-R) as an antigen, a human monoclonal stimulating autoantibody, M22, labeled with ruthenium as a competitor [13]. Immulite TSI (RRID:AB_2924340) manufactured by Siemens Healthcare Diagnostics (Llanberis, UK) consists of an automated chemiluminescent bridge-based assay that detects predominantly TSI using thyrotropin receptor chimeras [3, 4]. This test was calibrated according to the 2nd NIBSC (08/204), with a detection range of 0.1 to 40 IU/L; the optimal cutoff of 0.55 IU/L suggested GD diagnosis [3, 4]. Repeatability and within-lab imprecision were evaluated for both methods according to the Clinical Laboratory Standard Institute (CLSI) guideline EP15-A3 [14]. A total of 25 replicates in 5 days were performed using low and high levels of quality control reagents.

### Radioiodine Therapy

All patients were instructed to follow a low-iodine diet and to stop taking ATDs for at least 7 days before thyroid scintigraphy and uptake. Uptake was performed at 2 and 24 hours after oral administration of ^131^I (0.37 MBq) and scintigraphy was performed after intravenous administration of ^99m^Tc-sodium pertechnetate (370 MBq). Thyroid volume was estimated by routine ultrasound using the ellipsoid formula (volume = π/6×height [cm] × length [cm] × width [cm]). The activity of RAI administered was calculated to achieve an absorbed dose by the thyroid bed of 300 Gy in patients with GD. Dosimetry was carried out according to the Medical Internal Radiation Dose methodology and calculated according to our institution's protocol [15].

Refractoriness to RAI therapy was defined as the persistence or recurrence of a hyperthyroidism state after 6 months in the absence of levothyroxine treatment [6].

### Statistical Analysis

Statistical analysis was carried out using SPSS version 26.0.0.0 (IBM Corp, 2019) and JAMOVI version 2.3 (Jamovi Project, 2023). Categorical variables were presented as number and percentage. Mean and SD were calculated for continuous variables with normal distribution. Median and interquartile range (IQR p25-p75) were reported for continuous variables with non-normal distribution using the Shapiro–Wilk test and the Mann–Whitney U-test for comparison. For quantitative comparison between Elecsys TRAb and Immulite TSI, Spearman's correlation, Passing–Bablok regression and Bland–Altman analysis were calculated. Concentrations below the lower detection limit and over the upper detection limit of each method were considered equal to the limit values for statistical purposes. *P* < .05 was defined as statistically significant. For the prognostic analysis, logistic regression was calculated to define independent predictor variables of refractoriness to RAI treatment. Variables with multicollinearity were evaluated using principal component analysis to select the variable that accounted for the greatest proportion of the total variance explained by each component, and subsequently included in the generalized estimating equations. Generalized estimating equations is a robust method used to analyze repeated measures and correlated observations within the same individual, comparing variations in laboratory measurements taken before and at 3, 6, and 12 months after RAI therapy, using the baseline measurement as the reference point for the method. This approach helps to elucidate the clinical outcomes of patients with GD 1 year after RAI therapy. The sample size estimated a priori, for a type I error of 5%, power of 80% (type II error of 20%), and an effect size of ω = 0.3 (medium), was 143 patients (critical χ² = 11.0705), calculated using the G*Power program, version 3.1.9.4. Receiver operating characteristic curves were plotted and analyzed using SPSS version 26.0.0.0 (IBM Corp, 2019) to compare the area under the curve (AUC) by both methods.

## Results

### Patients’ Characteristics Before RAI Therapy

A total of 188 patients were included and prospectively studied, being 156 with GD and 32 with TNG. Their characteristics before RAI therapy are shown in Table 1. Age at RAI therapy ranged from 19 to 91 years. Dermatopathy was diagnosed in only 6 patients with GD and mild clinically inactive GO in 46.2% (n = 72). Prophylaxis with corticosteroids (0.3 mg/kg/day of prednisone) was administered to 20 patients with mild GO and to 2 patients without orbitopathy who had risk factors for development of orbitopathy (smokers or those with very high levels of Immulite TSI and/or Elecsys TRAb).

**Table 1.** Characteristics between Graves disease and toxic nodular goiter before RAI treatment

	Graves disease (n = 156)	Toxic nodular goiter (n = 32)	*P*
Age at RAI treatment (years)*^a^*	46.4 ± 13.1	58.0 ± 16.5	<.001*^c^*
Females (%)	122 (78.2%)	29 (90.6%)	.11
BMI (kg/m^2^)*^a^*	26.8 ± 5.9	25.5 ± 5.5	.22
Smoking, yes (%)	38 (24.4%)	9 (28.1%)	.78
Thyroid volume (cm^3^)*^b^*	24.5 (16.0-42.5)	28.1 (20.0-52.0)	.17
fT4 (pmol/L)*^b^*	38.5 (24.3-68.9)	18.8 (15.4-21.1)	<.001*^b^*
fT3 (pmol/L)*^b^*	10.8 (7.5-16.7)	6.6 (5.7-7.7)	<.001*^c^*
Elecsys TRAb (IU/L)*^b^*	6.0 (1.9-19.9)	0.39 (0.3-0.9)	<.001*^c^*
Immulite TSI (IU/L)*^b^*	4.0 (1.5-12.9)	0.1 (0.1-0.1)	<.001*^c^*
TPO-Ab (IU/mL)*^b^*	154 (14-563)	2 (2-3)	<.001*^c^*

Abbreviations: BMI, body mass index; fT3, free triiodothyronine; fT4, free thyroxine; RAI, radioactive iodine; TPO-Ab, thyroperoxidase antibody; TRAb, thyrotropin receptor autoantibody; TSI, thyroid-stimulating immunoglobulin.

^a^Mean ± SD.

^b^Median (IQR).

^c^ *P* < .05.

Failure to control hyperthyroidism and relapse after ATD trial (median of 35 months) were the main indications for RAI therapy in patients with GD (94.2%). Twelve patients received this treatment following adverse reactions to antithyroid medications (6 cases after significant cutaneous reactions, 2 due to neutropenia, and 1 due to agranulocytosis following methimazole, and 3 cases resulting from hepatopathy secondary to propylthiouracil).

### Precision

The repeatability for Immulite TSI in low-level samples (0.68 IU/L) and high-level samples (26.1 IU/L) were both 4.4%. The within-lab imprecision in low- and high-level samples were 2.6% and 5.6%, respectively. The repeatability for the Elecsys TRAb in low-level sample (1.99 IU/L) and high-level sample (30.7 IU/L) were 14.9% and 3.7%, respectively. The within-lab imprecision in low- and high-level concentrations were 7.3% and 1.9%, respectively.

### Comparison Between Elecsys TRAb and Immulite TSI

**Figure 1.**
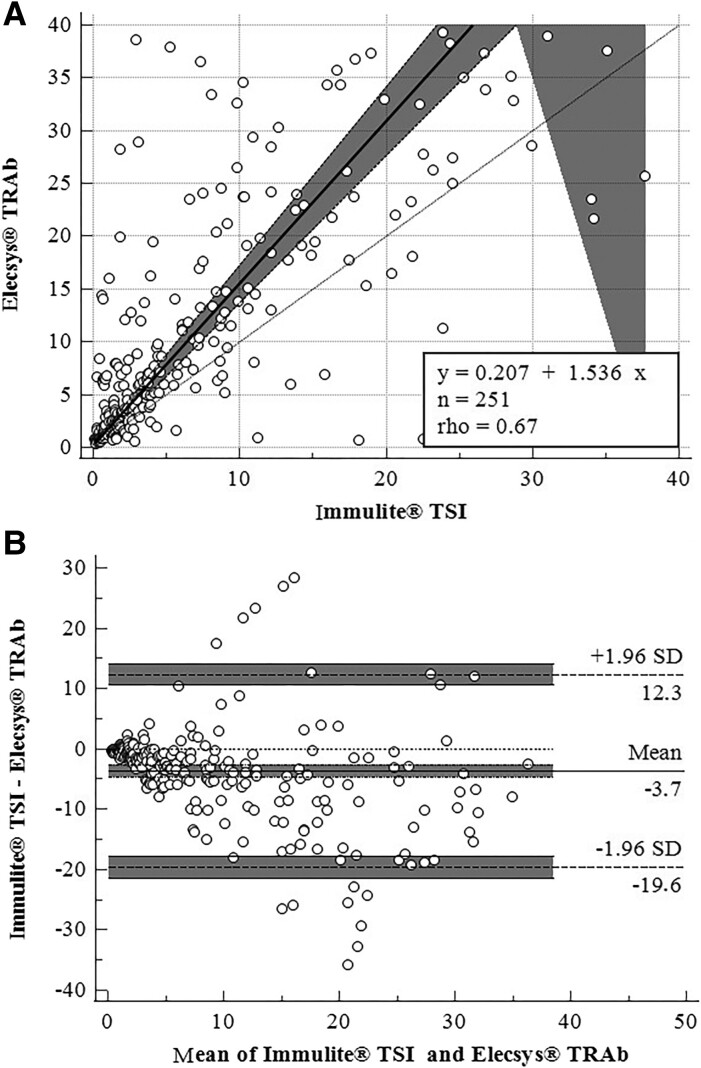
Correlation and agreement between Elecsys TRAbs and Immulite TSI. (A) Passing–Bablok regression. (B) Bland–Altman analysis.

In the total of 251 samples simultaneously collected and analyzed by Elecsys TRAb and Immulite TSI, the Spearman correlation showed positive linearity (rho = 0.67, *P* < .0001). The Passing–Bablok test showed an intercept of 0.207 (95% CI −0.212 to 0.405) and slope of 1.536 (95% CI 1.393-1.688), with no deviation from linearity (*P* = .25) (Fig. 1A). The Bland–Altman analysis showed acceptable agreement, with a mean bias of 3.7 IU/L (95% CI 2.6 to 4.7 IU/L) and confidence limits of −12.3 IU/L (95% CI −14.0 to −10.6) and 19.6 IU/L (95% CI 17.9 to 21.4) (Fig. 1B).

**Figure 2.**
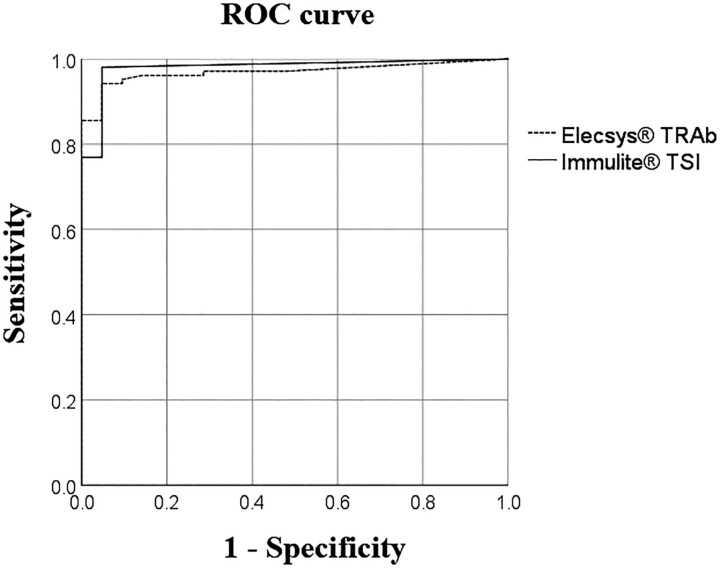
Receiver operating characteristic curve analysis comparing Elecsys TRAbs and Immulite TSI before radioactive iodine therapy.

The AUC for Immulite TSI and Elecsys TRAb did not show a statistically significant difference (*P* = .41) (Fig. 2). Using the cutoff values suggested by the manufacturers, the clinical sensitivity, specificity, and accuracy for GD diagnosis were, respectively, 92%, 96.2%, and 98.4% (AUC: 0.98) for Immulite TSI and 82.1%, 100%, and 91.2% (AUC: 0.97) for Elecsys TRAb.

### Prognosis of Successful RAI Therapy in Patients With Graves Disease

A total of 30 patients with GD were excluded from the study due to loss of follow-up, resulting in a final sample size of 126 patients. After 12 months of RAI therapy, successful treatment was achieved in 89% (112/126) of the patients with GD because hyperthyroidism was controlled without ATDs (3.2%) or caused hypothyroidism (85.8%). Novel RAI treatment was indicated in 3 patients, 7 patients resumed ATD treatment, and 4 patients maintained subclinical hyperthyroidism without medication. Considering patients with success and refractory to RAI therapy, both groups did not differ according to age, gender, previous duration or type of ATD treatment, smoking habits, TPO-Ab, or thyroglobulin antibody levels before RAI treatment (*P* > .05) (Table 2). Variables before RAI therapy associated with refractoriness 12 months after treatment of GD were thyroid volume, 2 hours of ^131^I thyroid uptake, thyroid absorbed dose (Gy), fT4 and fT3, and Elecsys TRAb and Immulite TSI concentrations (Table 2).

**Table 2.** Variables before RAI therapy associated with refractoriness 12 months after treatment of Graves disease

	Success (n = 112)	Refractory (n = 14)	*P*
Thyroid volume (cm^3^)*^a^*	23.7 (15.3-39.7)	37.2 (31.3-70.2)	<.001*^b^*
2 hours ^131^I thyroid uptake (%)*^a^*	37.0 (21.0-57.5)	64.0 (50.0-84.0)	.01*^b^*
RAI activity (mBq)*^a^*	592 (555-955)	610 (573-1147)	.28
Thyroid absorbed dose (Gy)*^a^*	430 (332-642)	347 (286-384)	.009*^b^*
fT4 (pmol/L)*^a^*	37.8 (23.4-66.8)	81.5 (34.8-100.0)	.007*^b^*
fT3 (pmol/L)*^a^*	10.8 (7.5-16.4)	21.8 (13.1-27.2)	.011*^b^*
Elecsys TRAb (IU/L)*^a^*	4.7 (1.9-13.3)	22.1 (2.5-27.7)	.04*^b^*
Immulite TSI (IU/L)*^a^*	3.3 (1.6-10.9)	8.1 (3.6-18.4)	.04*^b^*

Abbreviations: fT3, free triiodothyronine; fT4, free thyroxine; RAI, radioactive iodine.

^a^Median (IQR).

^b^ *P* < .05.

The therapeutic target of absorbed dose of ^131^I (≥300 Gy) was achieved in 71.4% of the refractory group and 85.7% of the therapeutic success group (*P* = .16). No statistically significant difference in the therapeutic outcome of GD was observed between cases that received >300 Gy or ≤300 Gy (90.2% and 81.0%, *P* = .19, respectively). Thyroid antibodies and fT3 compared before and 3, 6, and 12 months after RAI treatment were not related to refractoriness (n = 233 observations) (Table 3). For every 1 unit increase in Log_10_TSH (10 mIU/L), the risk of RAI refractoriness was reduced by an average of 112% (*P* < .05) (Table 3).

**Table 3.** Estimates of the fixed effects parameters, using the generalized estimated equations, in the prediction of refractoriness to RAI treatment in Graves disease

Variable independent	B	SE	OR (95% CI)	*P*
Log10 TSH	−0.92	0.38	0.40 (0.19-0.85)	.017*^a^*
fT3	−0.02	0.04	0.98 (0.91-1.06)	.90
Elecsys TRAb	0.06	0.05	1.06 (0.97-1.16)	.25
Immulite TSI	−0.05	0.10	0.95 (0.78-1.16)	.62
Elecsys TRAb * Immulite TSI	0.01	0.00	1.00 (0.99-1.01)	.70

Abbreviations: fT3, free triiodothyronine; RAI, radioactive iodine; TRAb, thyrotropin receptor autoantibody; TSH, thyrotropin; TSI, thyroid-stimulating immunoglobulin.

^a^ *P* < .05.

**Figure 3.**
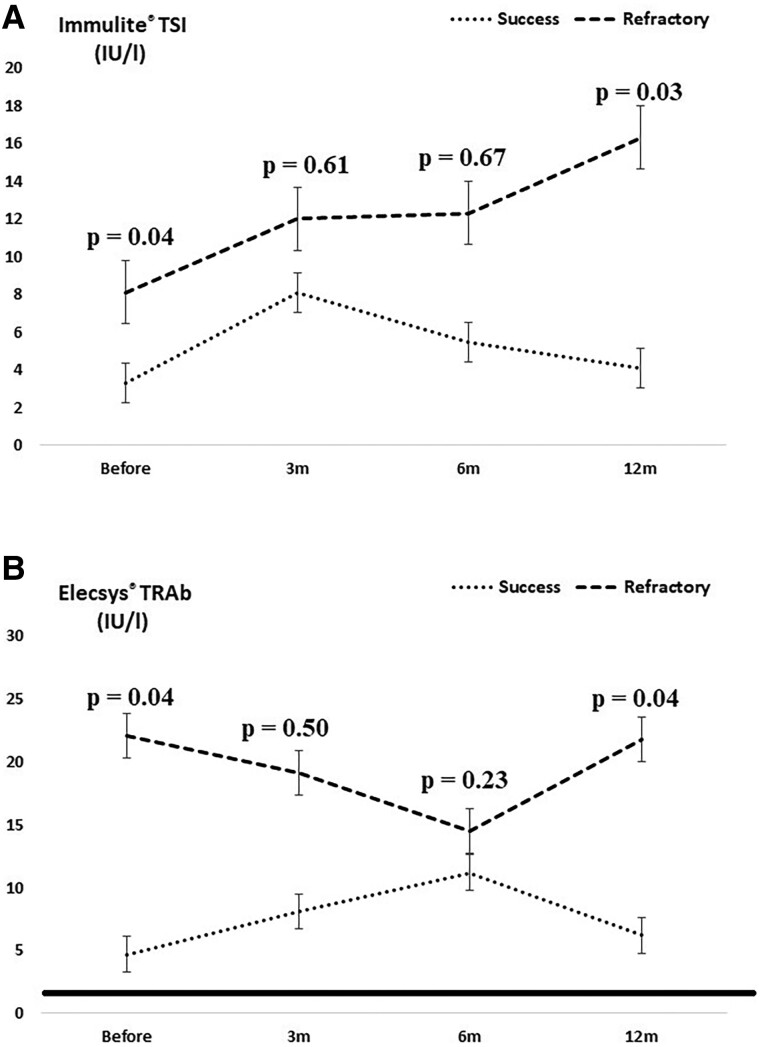
Variation in Immulite TSI (A) and Elecsys TRAbs (B) and before and after radioiodine therapy in Graves disease, according to therapeutic response. Th continuous line corresponds to Immulite TSI < 0.55 IU/L (A) and Elecsys TRAb < 1.75 IU/L.

As expected, both Elecsys TRAb and Immulite TSI levels increased after RAI treatment, with a later peak of Elecsys TRAbs (at 6 months) than Immulite TSI (at 3 months), and with progressive increment in the refractory group (Fig. 3A and 3B). It was noteworthy that high levels of Elecsys TRAb and Immulite TSI were still detected at the end of 12 months in most patients, 3 times above the cutoff at 53.1% and 65.8%, respectively.

## Discussion

TRAb and/or TSI determination is the recommended approach for the differential diagnosis of thyrotoxicosis, besides monitoring and prognosis of GD using ATD, and particularly in pregnant women with GD to assess the risk of complications to the fetus and neonate [6, 8]. In this prospective Brazilian cohort, we analyzed the prognostic value of Immulite TSI determination in patients with GD submitted to RAI therapy in comparison to Elecsys TRAb measurements. Elecsys TRAb is the leading automated assay in most Brazilian laboratories and abroad, and thus represents the most appropriate comparison for our purposes [16].

The results of Immulite TSI measurements demonstrated less variability at low concentrations than Elecsys TRAb measurements. As observed in previous studies, both assays showed good repeatability and within-lab variability [17, 18]. Accuracy at lower concentrations is crucial for the differential diagnosis of thyrotoxicosis, either as GD at its earliest stages or during the postpartum period, and also in patients with atypical ophthalmopathy [19]. The majority of patients enrolled in this study had a definitive diagnosis of GD and had already initiated ATD treatment but it had failed, which can result in different levels of circulating thyroid antibodies, irrespective of the methodology employed. Nevertheless, the accuracy at low concentrations of Immulite TSI could be useful for determining the remission of GD [6, 8].

By Bland–Altman analysis, both methods showed good agreement (bias: 3.7 IU/L, limits of agreement between −12.3 IU/L and 19.6 IU/L), but only a moderate correlation (rho = 0.67, *P* < .0001). Previous studies also compared these 2 methods but for diagnosis of GD and found higher correlation coefficients (R^2^ = 0.98) [20] and rho = 0.8099 [21]. However, based on the CI of the slope (95% CI 1.393 to 1.688), direct comparison and interchangeability between these 2 assays are not feasible [20]. This is not surprising at first, since the Immulite TSI, using a chimeric bridging interaction construct, is proposed to measure predominantly the stimulatory form of TRAb [3], while the Elecsys TRAb assay measures the total pool of antibodies present in the serum, TSI and TBII [13]. In addition, there are differences in the calibration of the methods used. The Immulite TSI was calibrated on the basis of the 2nd International Standard for Thyroid Stimulating Antibody, based on stimulating monoclonal antibodies of a well-defined concentration and nature, while Elecsys TRAb was calibrated on the basis of the 1st International Standard, which used a combination of stimulating and inhibiting immunoglobulins extracted from the plasma of a pregnant woman [22]. According to the cutoff proposed by the manufacturers, both methods showed a good AUC, but Immulite TSI showed a greater clinical sensitivity than Elecsys TRAb (92.0% and 82.1%, respectively) and clinical accuracy (98.4% and 91.2%, respectively) in the assessment of patients with GD before undergoing RAI therapy. The sensitivity of Immulite TSI was reported to be higher at 100%, because it was determined in untreated patients with GD [20, 23, 24]. Our findings revealed that in 11 out of 12 cases with inconsistent results among the methods (data not shown), the result consistently favored that indicated by Immulite TSI. This aligns with the results of another study, in which 10 out of 13 inconsistent results were more compatible with that indicated by Immulite TSI than Elecsys TRAb [17].

RAI therapy is an excellent choice when ATDs failed or caused severe side effects [6]. In our cohort, RAI treatment was indicated in 94% after failure with ATDs, even after a long period of use (mean of 35 months). Although thyroidectomy presents lower relapse rates (<10%), most clinicians still prefer RAI treatment to treat hyperthyroidism with excellent control [1]. Larger thyroid volume and severe hyperthyroidism are well-demonstrated prognostic factors for RAI treatment outcomes [6, 8, 25]. These findings were also observed in our study, where the thyroid volume in the refractory group was 50% larger (37.2 cm³ and 23.7 cm³, respectively; *P* < .001), and the levels of fT4 and fT3 were more than double those observed in the therapeutic success group (*P* < .05), although both groups had discontinued ATDs at the same time (at least 7 days prior to RAI therapy), which reflects more severe hyperthyroidism in these patients.

Fixed radioiodine activity of 185, 370, or 555 MBq or calculated radioiodine activity based on thyroid gland size and percentage uptake on scintigraphy demonstrated good successful outcome [15, 26]. Although equivalent activities of ^131^I were given to patients with GD, the refractory group in the present study received a lower radioiodine activity dose (347 Gy × 430 Gy, *P* = .009), indirectly reflecting the greater thyroid volume and uptake in these patients, probably related to a higher concentration of stimulating immunoglobulin. When considering radioiodine activity administered, there were no statistically significant differences in the outcomes between patients who received >300 Gy or ≤300 Gy. In a systematic review and meta-analysis that included 2303 patients with GD, a strong association was found between radiation absorbed dose and outcomes, with higher rates of euthyroid outcome with radiation absorbed doses within the range 120 to 180 Gy (n = 1172, OR 2.50, CI 1.17-5.35, *P* = .018) [27]. Euthyroidism is the best outcome, with lower calculated radioiodine activity [28], but follow-up longer than 12 months is needed to establish for how long the euthyroidism status would be sustained.

It has been demonstrated that higher systematic TRAb and TSI concentrations are associated with poor prognosis of ATD treatment and with higher risk for GO after RAI treatment, but showing contradictory results for RAI therapy outcomes [24, 29]. We demonstrated in our prospective evaluation that both Immulite TSI (*P* = .04) and Elecsys TRAb (*P* = .045) levels before treatment were independent predictors of therapeutic success of RAI treatment. Two previous studies using first-generation bioassays also highlighted that TSI values before RAI therapy were higher in patients who persisted in hyperthyroidism after treatment than in the therapeutic success group [11, 30]; the evidence regarding TRAbs is variable, with some studies showing higher levels of pretreatment TRAb among refractory patients [31, 32], whereas a recent meta-analysis found no differences in these results [33].

Finally, we analyzed whether longitudinal monitoring of laboratory variables could predict the therapeutic response to RAI treatment in patients with GD at the end of 12 months of follow-up. Only TSH variation contributed to this prediction, in which the risk of refractoriness to RAI therapy is estimated to decrease by an average of 9.2% for every 1 mIU/L increase in serum TSH. The therapeutic success group showed an increase in TSH in the first 3 to 6 months of follow-up, reflecting the occurrence of post-RAI hypothyroidism in 85% of cases. Unlike a previous study, which associated a greater increase in TRAb in the third month after RAI therapy within the group with hypothyroidism 1 year after RAI [34], the serial assessments of the Immulite TSI or Elecsys TRAb were not found to influence the prognosis of RAI therapy, despite the observed differences between the groups from the sixth month onwards. This ﬁnding represents an area that could be further investigated in future studies. It was also noteworthy that high levels of Elecsys TRAb and Immulite TSI were still detected at the end of 12 months in most patients, considered to be 3 times above the cutoff in 53.1% and 65.8%, respectively. After RAI treatment, pregnancy and breastfeeding are forbidden for 6 months and conception should be suspended for a period of 12 months. Our data showed high concentrations of both TSI and TRAb that will take more than 12 months to decrease and ensure a low risk to women of fertile age if they become pregnant [35].

Our study has several strengths. It is a prospective study with many individuals with GD who collaborated throughout the data collections, as well as the simultaneous assessment of 2 important TSH receptor antibodies assays, commonly available worldwide, reducing repeatability and within-lab imprecisions. However, it is important to note that there are also some limitations to be highlighted. Firstly, there is a risk of selection bias. Both the DG group and the TNG group, included in the calculations of clinical specificity, were recruited by referral to the Nuclear Medicine Service at our hospital. Therefore, it is important to be aware of this when interpreting the results. The study follow-up was 1 year, which needs to be longer particularly to evaluate risk of GO after RAI treatment and during pregnancy. Furthermore, a 20% loss to follow-up was observed among patients with GD. This represents a limitation of our study, as it may introduce bias and reduce the generalizability of the findings. Further studies with a larger sample size and improved retention rates would be valuable in validating these results and providing more robust conclusions.

In conclusion, Immulite TSI and Elecsys TRAb demonstrated similar clinical performance in patients with GD who required RAI therapy after failing clinical treatment. However, Immulite TSI showed less variability at low concentrations and exhibited higher clinical sensitivity and accuracy. Increased thyroid volume and uptake, along with higher levels of fT3 and fT4 before RAI, were also associated with a greater risk of refractoriness. Except for TSH variation, longitudinal monitoring of autoantibodies did not aid in predicting response to RAI after 12 months, though it remains important to track them due to their persistent high concentrations over time.

## Data Availability

Some or all datasets generated during and/or analyzed during the current study are not publicly available but are available from the corresponding author on reasonable request.

## Abbreviations

ATD: antithyroid drug
AUC: area under the curve
fT3: free triiodothyronine
fT4: free thyroxine
GD: Graves disease
GO: Graves orbitopathy
RAI: radioactive iodine
TBII: thyrotrophin-binding inhibiting immunoglobulin
TNG: toxic nodular goiter
TPO-Ab: thyroperoxidase antibody
TRAb: thyrotropin receptor antibody
TSI: thyroid-stimulating immunoglobulin
TT: total thyroidectomy
